# A Case of Advanced Glaucoma with Increased Episcleral Venous Pressure in a 17-Year-Old with Eisenmenger Syndrome

**DOI:** 10.1155/2017/5808047

**Published:** 2017-10-23

**Authors:** Leanne Grech, Adrian Mifsud, Maryanne Caruana, Francis Carbonaro

**Affiliations:** Mater Dei Hospital, Msida, Malta

## Abstract

Eisenmenger syndrome refers to reversal of shunt and central cyanosis due to pulmonary hypertension induced by congenital heart disease with a large systemic-to-pulmonary shunt. We report a case of a 17-year-old man with Eisenmenger syndrome who presented with gradual deterioration in visual acuity and was diagnosed with advanced secondary open angle glaucoma. There have been reports of patients suffering from thrombosis due to hyperviscosity associated with this syndrome; however, to our knowledge, the association of secondary open angle glaucoma with Eisenmenger syndrome has not yet been documented.

## 1. Introduction

Glaucoma is the leading cause of irreversible blindness worldwide [1] (Quigley, BJO, 1996). It can be classified into primary and secondary glaucoma. Pulmonary arterial hypertension is a known, but uncommon, cause of secondary open angle glaucoma, resulting from resistance to outflow of aqueous humour through the episcleral venous pathway. Eisenmenger syndrome refers to reversal of shunt and central cyanosis due to pulmonary hypertension induced by congenital heart disease associated with a large systemic-to-pulmonary shunt.

## 2. Case Presentation

A 17-year-old man presented with gradual deterioration in his vision. He was born with dextrocardia, double-outlet right ventricle, large perimembranous ventricular septal defect, and significant subvalvular pulmonary stenosis (PS). A palliative left Blalock-Taussig shunt was performed at the age of 2 years but no corrective surgery was possible as PAH ensued in the subsequent months, eventually leading to the development of Eisenmenger syndrome.

**Figure 1:**
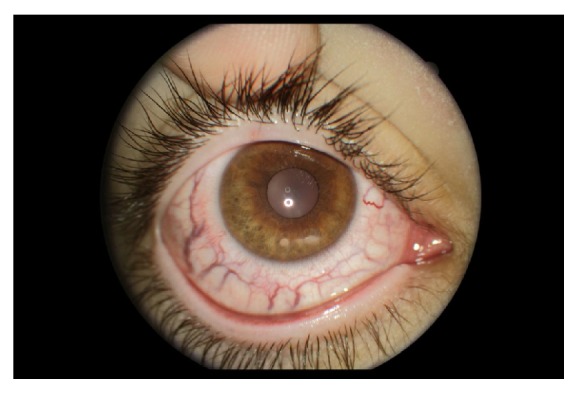
Right eye showing abnormally dilated episcleral vasculature.

**Figure 2:**
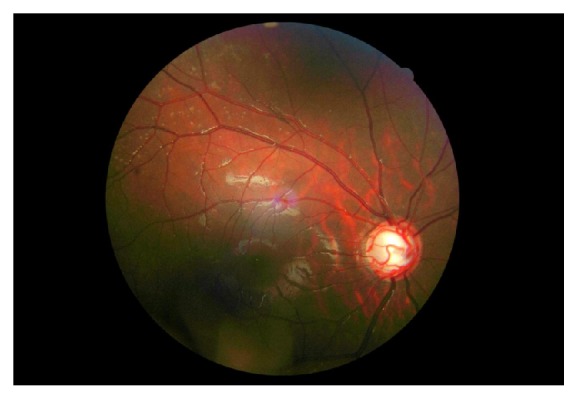
Right eye showing advanced optic disc cupping and glaucomatous looking optic nerve.

**Figure 3:**
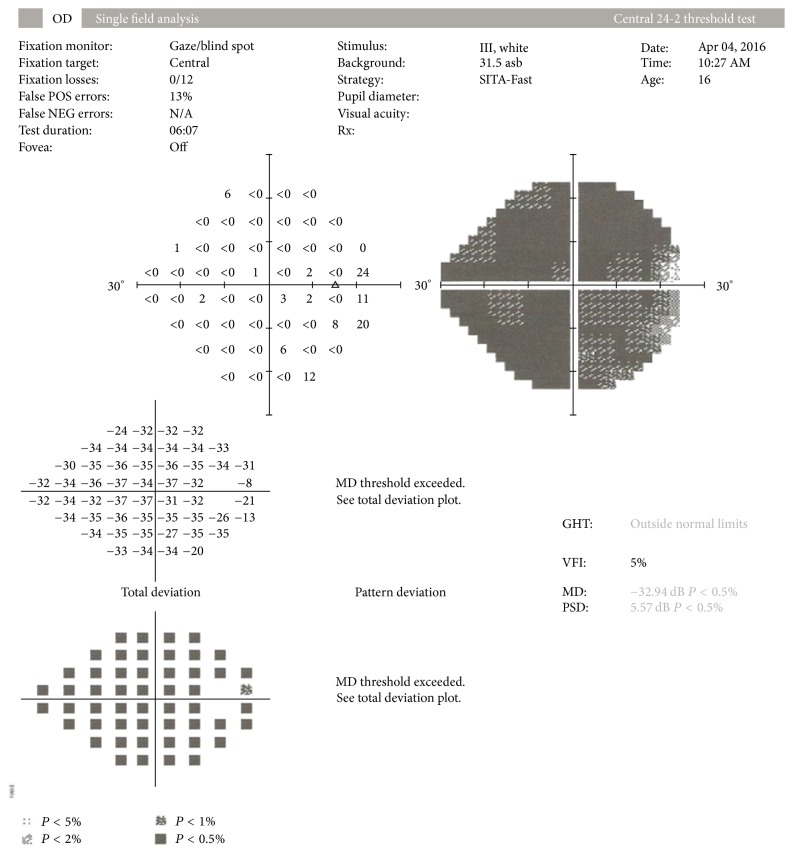
Humphrey's visual field analysis showing advanced glaucomatous visual field loss in his right eye.

On ophthalmic examination, visual acuity in his right eye was 6/10 and left eye 6/6. A striking appearance of dilated episcleral vasculature was seen in both eyes (Figure 1). A relative afferent pupillary defect was also present in his right eye. Intraocular pressure was found to be 14 mmHg in his right eye and 18 mmHg in the left eye. Fundoscopy revealed bilateral advanced optic disc cupping, consistent with glaucoma (Figure 2). Humphrey's visual fields test showed an advanced glaucomatous visual field defect in his right eye (Figure 3) and an early superior arcuate defect in his left visual field. A diagnosis of advanced secondary open angle glaucoma was made and he was commenced on latanoprost drops in both eyes, following which intraocular pressure decreased to 8 mmHg in right eye and 10 mmHg in left eye. He is being monitored on a regular basis.

## 3. Discussion

Glaucoma is a chronic progressive optic neuropathy, which results in irreversible damage to the optic nerves with characteristic optic nerve changes and characteristic visual field loss. Unfortunately, at the time of presentation this patient's glaucoma was already quite advanced, especially in his right eye. The mechanism behind this case is thought to be due to increased episcleral venous pressure over many years. This type of secondary glaucoma in the setting of pulmonary hypertension, with dilated episcleral veins, has been documented before [2–4] (Parikh et al., 2011; Radius and Maumenee, 1978; Keltner et al., 1987). The pulmonary hypertension results in elevated episcleral venous pressure which hinders the drainage of aqueous humour across the trabecular meshwork and into the blood supply, thus resulting in elevated intraocular pressure (IOP). His IOP levels were not that severely elevated at presentation, with most of the damage having probably occurred at previous times when the pulmonary arterial pressure was more elevated. There have been previous reports of patients suffering from thrombosis due to hyperviscosity associated with this syndrome, including an interesting case report in 2006 [5–7] (Mathura Jr. and Jampol, 2006; Rishi et al., Ind Journ Ophthl 2010; Rodriguez and Eliott, AJO 2001); however, to our knowledge, the association of glaucoma with increased episcleral venous pressure and Eisenmenger's syndrome has not as yet been documented. The late presentation of this case and the absence of signs such as Haab striae and increased corneal diameter differentiate it from congenital glaucoma.

The patient is now controlled well on prostaglandin analogue drops and he is being kept under regular review with regular visual field tests and Ocular Coherence Topography scans.

We hope that this case will bring this possible association of secondary glaucoma with Eisenmenger syndrome to light. We recommend that patients with this cardiac abnormality have regular ophthalmic checks, to rule out glaucoma.

## References

[1] Quigley H. A. (1996). Number of people with glaucoma worldwide. *British Journal of Ophthalmology*.

[2] Parikh R. S., Desai S., Kothari K. (2011). Dilated episcleral veins with secondary open angle glaucoma. *Indian Journal of Ophthalmology*.

[3] Radius R. L., Maumenee A. E. (1978). Dilated episcleral vessels and open-angle glaucoma. *American Journal of Ophthalmology*.

[4] Keltner J. L., Gittinger Jr. J. W., Miller N. R., Burder R. M. (1987). A red eye and high intraocular pressure. *Survey of Ophthalmology*.

[5] Mathura J. R., Jampol L. M. (2006). Medical mystery: visual-field defect—the answer. *The New England Journal of Medicine*.

[6] Rishi P., Rishi E., Sharma T., Mahajan S. (2010). Hemi-central retinal artery occlusion in young adults. *Indian Journal of Ophthalmology*.

[7] Rodriguez N., Eliott D. (2001). Bilateral central retinal vein occlusion in Eisenmenger syndrome. *American Journal of Ophthalmology*.

